# Mutations, Genes, and Phenotypes Related to Movement Disorders and Ataxias

**DOI:** 10.3390/ijms231911847

**Published:** 2022-10-06

**Authors:** Dolores Martínez-Rubio, Isabel Hinarejos, Paula Sancho, Nerea Gorría-Redondo, Raquel Bernadó-Fonz, Cristina Tello, Clara Marco-Marín, Itxaso Martí-Carrera, María Jesús Martínez-González, Ainhoa García-Ribes, Raquel Baviera-Muñoz, Isabel Sastre-Bataller, Irene Martínez-Torres, Anna Duat-Rodríguez, Patrícia Janeiro, Esther Moreno, Leticia Pías-Peleteiro, Mar O’Callaghan Gordo, Ángeles Ruiz-Gómez, Esteban Muñoz, Maria Josep Martí, Ana Sánchez-Monteagudo, Candela Fuster, Amparo Andrés-Bordería, Roser Maria Pons, Silvia Jesús-Maestre, Pablo Mir, Vincenzo Lupo, Belén Pérez-Dueñas, Alejandra Darling, Sergio Aguilera-Albesa, Carmen Espinós

**Affiliations:** 1 Rare Neurodegenerative Diseases Laboratory, Centro de Investigación Príncipe Felipe (CIPF), 46012 Valencia, Spain; 2 Joint Unit CIPF-IIS La Fe Rare Diseases, 46012 Valencia, Spain; 3 Paediatric Neurology Unit, Department of Paediatrics, Hospital Universitario de Navarra, Navarrabiomed, 31008 Pamplona, Spain; 4 Structural Enzymopathology Unit, Instituto de Biomedicina de Valencia (IBV), Consejo Superior de Investigaciones Científicas (CSIC), Centro de Investigación Biomédica de Enfermedades Raras (CIBERER-ISCIII), 46010 Valencia, Spain; 5 Biodonostia Health Research Institute, Paediatric Group, Donostia University Hospital, Department of Paediatrics, University of the Basque Country UPV/EHU, 20014 San Sebastian, Spain; 6 Paediatric Neurology Unit, Hospital Universitario Cruces, 48903 Barakaldo, País Vasco, Spain; 7 Health Research Institute, Hospital Universitari i Politècnic La Fe, 46026 Valencia, Spain; 8 Movement Disorders Unit, Neurology Department, Hospital Universitari i Politècnic La Fe, 46026 Valencia, Spain; 9 Paediatric Neurology Unit, Hospital Infantil Universitario Niño Jesús, 28009 Madrid, Spain; 10 Centro de Referencia de Doenças Hereditarias do Metabolismo, CHULN, Hospital Santa Maria, 1649-035 Lisbon, Portugal; 11 Department of Paediatrics, Hospital Regional Universitario, 29010 Malaga, Spain; 12 Paediatric Neurology Unit, Hospital Sant Joan de Déu, 08950 Barcelona, Spain; 13 Department of Paediatrics, Hospital Universitari Son Espases, 07120 Palma de Mallorca, Spain; 14 Unit of Parkinson and Movement Disorders, Department of Neurology, Hospital Universitari Clínic, 08036 Barcelona, Spain; 15 Paediatric Neurology, Hospital Agia Sofía, 11527 Athens, Greece; 16 Movement Disorders Unit, Department of Neurology and Clinical Neurophysiology, Instituto de Biomedicina de Sevilla, Hospital Universitario Virgen del Rocío/CSIC/Universidad de Sevilla, and Centro de Investigación Biomédica en Red de Enfermedades Neurodegenerativas (CIBERNED-ISCIII), 41013 Seville, Spain; 17 Department of Paediatric Neurology, Hospital Universitari Vall d’Hebron, Vall d’Hebron Institut de Recerca, 08035 Barcelona, Spain; 18 Department of Biotechnology, Faculty of Veterinary and Experimental Sciences, Universidad Católica de Valencia “San Vicente Mártir”, 46001 Valencia, Spain

**Keywords:** movement disorders, ataxia, cerebellar atrophy, neurodegeneration with brain iron accumulation (NBIA), gene panel, exome sequencing

## Abstract

Our clinical series comprises 124 patients with movement disorders (MDs) and/or ataxia with cerebellar atrophy (CA), many of them showing signs of neurodegeneration with brain iron accumulation (NBIA). Ten NBIA genes are accepted, although isolated cases compatible with abnormal brain iron deposits are known. The patients were evaluated using standardised clinical assessments of ataxia and MDs. First, NBIA genes were analysed by Sanger sequencing and 59 patients achieved a diagnosis, including the detection of the founder mutation PANK2 p.T528M in Romani people. Then, we used a custom panel MovDisord and/or exome sequencing; 29 cases were solved with a great genetic heterogeneity (34 different mutations in 23 genes). Three patients presented brain iron deposits with Fe-sensitive MRI sequences and mutations in *FBXO7*, *GLB1*, and *KIF1A*, suggesting an NBIA-like phenotype. Eleven patients showed very early-onset ataxia and CA with cortical hyperintensities caused by mutations in *ITPR1*, *KIF1A*, *SPTBN2*, *PLA2G6*, PMPCA, and *PRDX3*. The novel variants were investigated by structural modelling, luciferase analysis, transcript/minigenes studies, or immunofluorescence assays. Our findings expand the phenotypes and the genetics of MDs and ataxias with early-onset CA and cortical hyperintensities and highlight that the abnormal brain iron accumulation or early cerebellar gliosis may resembling an NBIA phenotype.

## 1. Introduction

In 2015, we initiated a new research line focused on disorders associated with neurodegeneration with brain iron accumulation (NBIA). This group of syndromes is characterised by movement disorders (MDs) with the common feature of abnormal deposits of iron in the brain, although clinical and neuroimaging features are variable, reflecting the genetic heterogeneity in this group [1,2,3]. Pantothenate kinase-associated neurodegeneration (PKAN), caused by *PANK2* mutations [4], is the most frequent NBIA phenotype; dystonia, parkinsonism, and cognitive decline over the years are core features. In *PLA2G6*-associated neurodegeneration (PLAN), the second most frequent NBIA, hypotonia, ataxia, and early progressive cerebellar atrophy (CA) are the hallmark of the disease, with brain iron deposits detected in only 50% or less of cases [5]. Different clinical characteristics are predominant in other NBIA disorders, as ataxia, spastic paraparesis, peripheral neuropathy, optic atrophy, and neuropsychiatric problems. Thus, the clinical phenotype and brain magnetic resonance imaging (MRI) patterns evolve differently in each disorder and depend on the stage of the disease and the age of the patient [3].

In clinical practice, genetic diagnosis in these phenotypes may end up being a cumbersome odyssey, due to the difficulty in making reliable genotype-phenotype correlations. Assessment of next generation sequencing (NGS) tools has been reported for cohorts with MDs [6], and more frequently for specific sets of entities such as ataxia [7,8] or hereditary spastic paraplegia (HSP) [9,10]. Targeted gene sequencing likely represents the most cost-effective option, whereas the gold standard seems to be whole exome sequencing (WES) as it covers the known coding genome. 

In our cohort, the sequencing of selected NBIA genes in 114 patients provided the genetic diagnosis of 59 patients, including the detection of the founder mutation PANK2 p.T528M in the Roma population. We then studied the undiagnosed patients using a custom panel MovDisord and WES-trio, plus 10 additional cases investigated exclusively by WES-proband. Twenty-nine probands achieved a conclusive molecular diagnosis with a great genetic heterogeneity (34 different disease-causing mutations in 23 genes). Considering the whole series, the diagnostic success rate was 70.97%. We expanded MDs phenotypes with brain iron accumulation, but also ataxia phenotypes with CA and cortical hyperintensities, from classical PLAN to non-progressive congenital ataxia (NPCA) phenotypes. 

## 2. Results

### 2.1. Genetics

**Figure 1:**
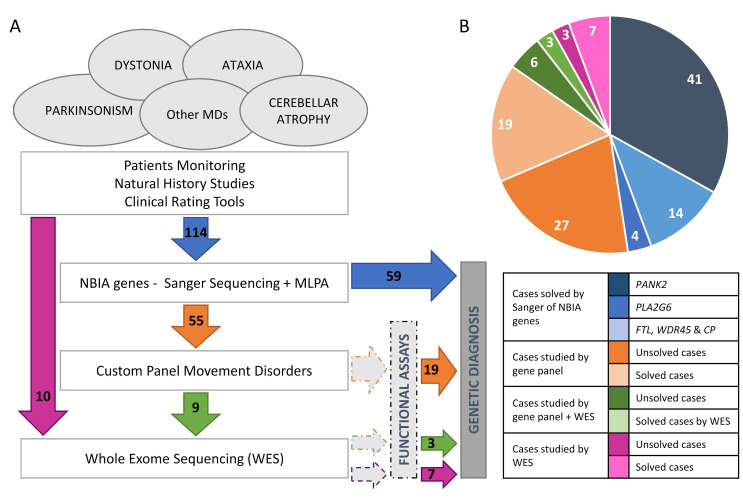
Casuistry and strategy. (**A**) Pipeline designed for genetic analysis of a cohort of patients with movement disorders and ataxia, which includes different approaches from candidate gene to gene panel and WES (whole exome sequencing) carried out in a cohort of 124 probands. (**B**) Distribution of cases studied by gene panel MovDisord. In 59 probands, a definitive diagnosis was achieved by Sanger sequencing and MLPA (in blue): 41 patients carried mutations in *PANK2*, 14 in *PLA2G6*, and 4 in other NBIA genes (http://espinos.cipf.es/index.php/en/mutations-db, accessed on 1 August 2022). Using a custom panel MovDisord, the remaining 55 probands were investigated and the causative mutations were identified in 19 cases (in orange). Finally, nine patients were further studied by WES and in three cases the clinical variant was detected (in green). To compare both approaches (gene panel *versus* WES), ten patients were investigated by WES only, and the disease-causing mutations were detected in seven of them (in purple). NBIA: neurodegeneration with brain iron accumulation. MD: Movement disorders. MLPA: multiplex ligation-dependent probe amplification.

The mutational screening of the most frequent NBIA genes revealed that 41 (35.96%) and 14 (12.28%) out of 114 patients carried biallelic mutations in *PANK2* or *PLA2G6*, respectively (Figure 1). Regarding other NBIA genes, one proband harboured a causative variant in *WDR45* (c.182A>C, p.N61T); two in *FTL* (c.286G>A, p.A96T; c.509_*4del, p.L170_Pfs*8); and another one in *CP* (c.1652C>T, p.T551I/c.2684G>C, p.G895A). Taken together, the etiological diagnosis was attained in 59 (51.75%) patients. The NBIA mutations detected in our series are available at https://espinos.cipf.es (accessed on 1 August 2022).

The seven unrelated Roma patients from Spain (homozygous for the *PANK2* c.1583C>T, p.T528M mutation), shared the same haplotype [A-268-C-T-C-G-285] that stretched from rs1078152 to *D20S116*, encompassing a 0.9 Mb region (Table S1). The Hungarian Roma proband had a narrower haplotype for both alleles, which included the intragenic markers (13.1 Kb) only. Two Greek heterozygous carriers of the *PANK2* c.1583C>T mutation presented the same haplotype as the Spanish patients, and one Greek proband showed a finer haplotype from rs1078152 to rs7270329 (0.8 Mb). Lastly, the non-Roma Spanish proband heterozygous for the investigated variant had a different haplotype, suggesting different mutational events for *PANK2* c.1583C>T. 

The 55 unsolved patients were investigated by a custom panel MovDisord (Figure 1). The mean coverage among all samples was >400×, the lowest mean coverage achieved was 233×, and the highest 605× (Figure S1). In all cases, at least 99.7% of the bases were covered with >20 reads and mean gene coverage was >65× (Table S2). Panel MovDisord was robust in terms of coverage, as well as read depth for all genes and samples.

Nineteen out of fifty-five patients were genetically diagnosed (Table 1 and Figure 1). Nine unsolved patients were then investigated by WES-trio and mutations in *PLEKHG2*, *NR4A2,* and *PRDX3* were identified in three probands (Table 1). None of these three genes were included in the panel. In parallel, seven out of ten patients, investigated by WES-proband only, obtained a conclusive genetic diagnosis (Table 1 and Figure 1). It is worth mentioning that the MD-319 case (*LRRK2* gene) was cleared up after a reanalysis of WES data two years after the initial analysis. In total, 29 patients were diagnosed. 

Two cases were familial and one case currently sporadic (MD-252), likely would be a familial one (the daughter’s proband is asymptomatic; Figure S2 and Table 2); twenty-five cases were sporadic (11 de novo cases); and one case remained unclassified due to lack of information. Most of the deleterious changes were transmitted in an AR fashion (16 cases) or in an AD manner (12 cases), and only one case presented an X-linked dominant (XLD) inheritance. Nine AR cases had mutations in homozygosis, and in seven of them, the parents were consanguineous (five patients from Morocco, one from Greece, and one from Spain). Two mutations were identified twice: EXOSC3 p.D132A in homozygosis in non-consanguineous cases, and KIF1A p.R316W with a de novo presentation (Table 1 and Table 2). As a whole, 34 mutations were detected, 17 of them not previously associated with disease, in 23 different genes, including those formerly published [12,13,14,15,16,17].

### 2.2. Genotype-Phenotype Correlation

**Figure 2:**
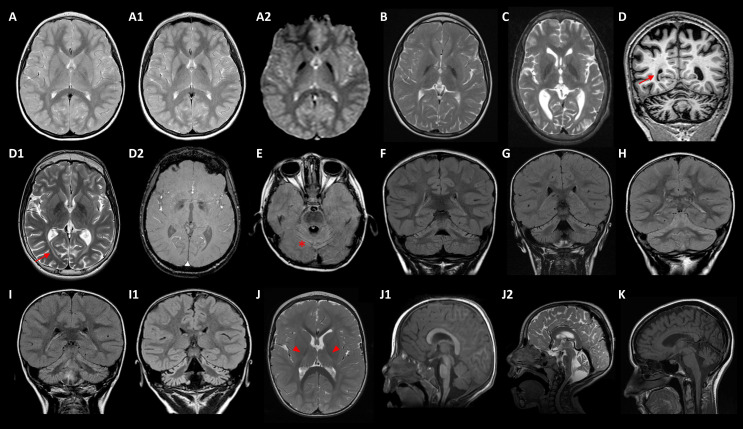
Main neuroimaging features. (**A**–**A2**) MD-020/*GLB1*: axial TSE (turbo spin echo) T2 weighted image at ages 6 (**A**) and 11 (**A1**) showing progression of *globus pallidus* (GP) hypointensities, demonstrating iron deposition in axial T2* GRE (gradient echo) image (**A2**) at 11 years old. (**B**) MD-137/*FUCA1* and (**C**) MD-153/*TPP1* revealing T2-weighted images (T2WI) GP hypointensities at ages 10 and 14, respectively. (**D**–**D2**) MD-189/*KIF1A* at 17 years old: coronal T1-weighted image showing cerebral and cerebellar atrophy (**D**), axial T2WI revealing GP hypointensity (**D1**) and SWI, susceptibility weighted imaging (**D2**) showing iron deposition; the patient also presented periventricular and deep white matter abnormal signal (arrows). (**E**) MD-200/*KIF1A* at age of 4: axial FLAIR (fluid attenuated inversion recovery) image revealing global cerebellar atrophy with cortical hyperintensity (* tram-track sign). (**F**–**H**) Coronal FLAIR images uncover predominantly upper cerebellar cortical hyperintensity in patients MD-106 at age of 3 (**F**), MD-041 of 4 (**G**), and MD-320 of 4 (**H**), with *ITPR1* variants. (**I**–**I1**) MD-323/*PMPCA*: Coronal FLAIR images at 2 years old (**I**), and at 8 years old (**I1**) demonstrating progression of cerebellar atrophy with cortical hyperintensities. (**J**–**J2**) MD-181/*PLEKHG2* from 2 (**J**,**J1**) to 5 years old (**J2**): T2WI (**J**) showing thalamic and posterior internal capsule hyperintensities (head arrows), and mild deep white matter hyperintensity; mid-sagittal T1 weighted image (**J1**) and midsagittal T2WI (**J2**) demonstrating progression of cerebellar atrophy. (**K**) MD-270/*HEXA*: mid-sagittal T2WI revealing cerebellar atrophy at disease onset (23 years old).

Five cases with T2W MRI showing bilateral GP hypointensities carried disease-causing mutations in *FBXO7*, *GLB1, FUCA1, TPP1,* and *KIF1A* (Table 1 and Table 2) MD-018 (homozygous FBXO7 p.S123*) was previously described [14]. MD-020 (GLB1 p.R59H/p.Y36C) suffers from a GM1-gangliosidosis type II, with an onset at age 3 with dysphemia. The patient showed a slow progression of the motor disorder, with generalized dystonia at age 12, associated with bulbar dysfunction (dysphagia, dysarthria, dysphonia), and mild flattening of vertebral bodies with scoliosis; iron-sensitive MRI sequences showed brain iron deposits from 10 years old (Figure 2A–A2). MD-137 (homozygous FUCA1 p.R47P) with a history of static encephalopathy, had a dystonic gait from 3 years old and presented GP T2W hypointensities from 10 years old (Figure 2B). MD-153 (TPP1 p.R447H/p.G77R) suffered from a progressive spastic-dystonic gait and also, showed T2W GP hypointensities, together with CA and deep white matter subtle demyelination, at the age of 14 (Figure 2C). Finally, MD-189 (KIF1A p.R316W) presented with an ataxic-spastic gait with early-onset CA, but iron deposits ascertained by SWI sequences from 17 years old (Figure 2D–D2).

Eleven diagnosed patients manifested very early-onset ataxia and CA associated with mutations in *ITPR1* (3), *KIF1A* (3), *PLA2G6, PMPCA, SPTBN2* (2), and *PRDX3* (Table 1 and Table 2). The patients with mutations in *SPTBN2* or *PRDX3* were previously reported [13,17]. Interestingly, nine subjects evolved to an NPCA phenotype [18]; they harboured mutations in *ITPR1, KIF1A, PMPCA,* and *SPTBN2*. Additionally, five patients with *ITPR1* (2) or *KIF1A* (3) variants developed lower limbs spasticity over time; two children with *KIF1A* mutations additionally associated optic atrophy, and one of them showed the early appearance of iron deposits demonstrated by iron-sensitive sequences (Figure 2D2). 

In the reported PLAN series [5], CA is universal and one-third of the patients showed cerebellar cortical hyperintensities, discovered by axial or coronal FLAIR (fluid attenuated inversion recovery) MRI sequences. Remarkably, this neuroimaging finding was also observed in seven out of nine NPCA patients (Figure 2E–I1), related to deleterious variants in *KIF1A* (1), *ITPR1* (3), *PMPCA* (1), and *SPTBN2* (2) [13]. MD-181 (homozygous PLEKHG2 p.T53I) showed very early-onset hypotonia, and developmental delay rapidly evolved into spastic-dystonic tetraparesis from 6 months old (Table 1 and Table 2), when subtle symmetrical T2 hyperintensities on *thalami* and deep white matter were detected (Figure 2J). The clinical course showed a significant deterioration with the appearance of oculomotor apraxia, peripheral neuropathy, and CA from 2 to 5 years old (Figure 2J1–J2). MD-159 (SPG7 p.A510V/p.A572V), previously reported [15,19], presented early onset multifocal dystonia with severe cranio-cervical involvement and mild CA on brain MRI. MD-270 (*HEXA* c.459+5G>A/c.1305C>T) started with a cerebellar syndrome at 23 years old with cognitive decline and CA (Figure 2K). MD-296 (homozygous QARS1 p.R265H) suffered from a developmental delay and seizures from the second year of age, associated with mild CA without ataxia.

Five patients developed a severe encephalopathy with a pontocerebellar hypoplasia (PCH) pattern (Table 1 and Table 2). MD-208 (heterozygous *CASK* c.2589+2T>G), a 5-year-old girl, also had congenital microcephaly with PCH and generalised CA. MD-307 (RPGR1P1L p.K233*/p.S590Cfs*), an 11-month boy, developed progressive encephalopathy with respiratory complications and early death. MD-307 and MD-216 (CPLANE1 p.S1127A/c.7588+3A>G) presented the characteristic molar tooth sign on brain MRI associated with Joubert syndrome. Lastly, two patients, MD-122 and MD-012 (homozygous EXOSC3 p.D132A), suffered from progressive encephalopathy with spasticity, muscular weakness, and early and rapid vermis atrophy. 

Only one case showed an MRI with calcium depositions: MD-173 (*PDGFB* c.602-1G>C) and her affected mother, who presented a mild phenotype with postural and intention tremors. The remaining four patients did not show any remarkable findings in brain MRI (Table 1 and Table 2). MD-126 (REEP1 arr[hg19]2p11.2 (chr2:83,335,425-87,271,924)) and MD-277 (NR4A2 p.R319Q) were previously reported [12,16]. MD-252 (*PNKD* c.-4C > G), presented at the age of 49 with paroxysmal episodes of lingual dystonia and blepharospasm. The attacks lasted for a few minutes and were usually precipitated after prolonged speaking. Further, mild upper limb dystonic postures were evident on examination. An extensive study including MRI and magnetic resonance angiography, electroencephalogram, dopamine transporter single photon emission CT (DaT-SPECT) and a thorough laboratory investigation, was performed without findings. Segregation analysis revealed that his healthy daughter, 25 years old, heterozygous for the *PNKD* c.-4C>G, remained asymptomatic (Figure S2). MD-319 (LRRK2 p.G2019S) started with tremors at age 5; by 14 years old, he presented bradykinesia and a hoarse voice.

A whole deletion of *NIPA1* was observed in heterozygosis in two patients, which was a heterozygous 15q11.2 BP1-BP2 microdeletion (Table 3) whose implications in disease are controversial [20]. Thus, MD-168 and MD-143 presented with disparate symptoms, and therefore, it is not possible to conclude that the 15q11.2 BP1-BP2 microdeletion is the only molecular cause underlying illness in our probands. 

MD-232 (heterozygous NIPA1 p.P91R) carried a duplication on chromosome 18 and a deletion on chromosome 18q 46,XY, der(18)del(18)(q22-qter)invdup(18)(p11.1-pter) (Table 3), with unclear clinical consequences. The patient showed T2W GP hypointensities, although the MRI sequences were not conclusive at age 11. Strikingly, the immunofluorescence assay displayed that NIPA1 p.P91R may alter the intracellular transport (Figure S3), but how it contributes to the final clinical outcome remains elusive.

MD-179 (heterozygous GCH1 p.K224R), MD-342 (homozygous PARK2 p.W74Cfs*), and MD-347 (SACS p.Q1143K/p.N4573H) carried interesting variants as candidates to be the disease-causing mutations (Table 3 and Figure S2). Nonetheless, the segregation analyses and/or the clinical picture made us discard them as causative mutations in these probands. 

### 2.3. Studies to Investigate the Pathogenicity of the Novel Variants 

In the 65 patients studied by gene panel MovDisord and/or WES, 17 novel causative variants were identified (Table 1). We performed additional studies to obtain evidence of pathogenicity. We previously reported the analyses of four novel mutations located in *FBXO7*, *SPTBN2*, and *PRDX3* [13,14,17]. GLB1 p.Y36C must be damaging because the affected girl showed an extremely low blood β-galactosidase activity (4 nmol/h per mg protein), which supports a defective GLB1 protein. RPGR1P1L p.S590Cfs* change is expected to be detrimental as it is a premature stop codon. Thus, we describe studies for nine novel variants, including a new change (NIPA1 p.P91R) detected in a patient with a controversial diagnosis (Table 3), which was studied to ascertain if it may contribute to the clinical outcome. 

**Figure 3:**
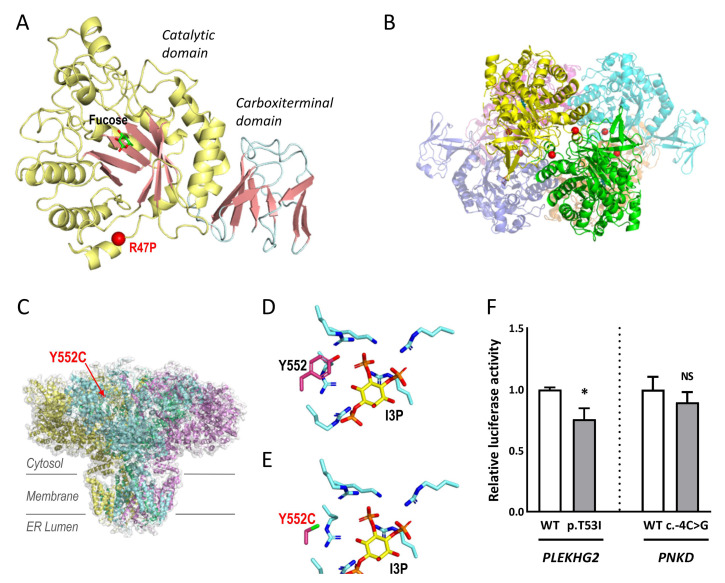
Structural modelling and luciferase assays. (**A**,**B**) FUCA1 p.R47P. (**A**) Structural modelling on HuFUCA1 subunit. The catalytic and carboxy terminal domains are coloured differently. Localization of R47P is mapped with a red sphere. A molecule of fucose bound to the active site is shown in sticks representation. (**B**) Hexamer of TmFUCA1 (PDB 1ODU). Each subunit is shown with a different colour. The equivalent residue to R47 from HuFUCA1 is indicated by red spheres, to show its participation in the intersubunit surface. (**C**–**E**) ITPR1 p.Y552C: (**C**) The cryo-EM structure of ITPR1 tetramer (PDB 7LHE) is represented in an orientation along the membrane plane. Each subunit is shown with a different colour. Y552 is mapped in one of the subunits with a red sphere and labelled. (**D**,**E**) Detail of the IP3 binding site (**D**), PDB 1N4K, and modelling of the Y5552C variant (**E**). The side chain of tyrosine or cytosine at position 552 as well as those from the lysine/arginine cluster coordinating the phosphoryl groups of I3P are shown in stick representation. I3P is also shown in stick representation with carbon atoms coloured in yellow. Oxygen, nitrogen, phosphorus, and sulphur atoms are coloured red, blue, orange, and green respectively. (**F**) Luciferase assays to assess PLEKHG2 p.T53I and *PNKD* c.-4C>G variants in transfected HEK293T with expression constructs for wild-type (WT) and mutants. The luciferase activity was normalised to the WT. Data represent the mean ± SEM of three independent experiments performed in triplicate. * *p* < 0.05; n.s., not significant.

FUCA1 p.R47P, and ITPR1 p.Y552C were explored by structural modelling. Importantly, the proband MD-137 showed a complete absence of α-fucosidase activity (0.00 nmol/h per mg protein), supporting that the patient suffered from a α-fucosidase deficiency. Tissue α-L-fucosidase (FUCA1) is a lysosomal enzyme responsible for hydrolysing the α-1,6-linked fucose of fucose-containing glycoproteins and glycolipids. Despite its clinical importance, the three-dimensional structure of human or other animal FUCA1 is not known. However, the high percentage of sequence identity (35%) with FUCA1 from *Thermotoga maritima* (TmFUCA1) [21] allows reliable modelling of human FUCA1 (HuFUCA1; Figure 3A), which is practically identical to the model generated by AlphaFold (rmsd 0.932 Å/298 Cα) [22]. R47 is localised at the catalytic domain of HuFUCA1, away from the active site, in the N-terminal α-helix, that in TmFUCA1 mediates the interactions between subunits of the molecular hexamer (Figure 3B). Sequence conservation between TmFUCA and HuFUCA1 sequences does not imply that they have equivalent architectures. Therefore, we cannot anticipate whether or not R47 will play a key role in maintaining the stability of the oligomer. What is certain is that the R47P mutation is not compatible with maintaining the amino-terminal helix of FUCA1. It is therefore predictable that the mutation may disrupt the helix, probably affecting the correct folding of the subunit, and consequently its stability, which may support the absence of α-fucosidase activity in the patient.

The inositol 1,4,5-trisphosphate (IP3) receptor (ITPR1), a tetrameric transmembrane channel located in the endoplasmic reticulum membrane (Figure 3C–E) [23], releases Ca^2+^ to the cytosol in response to IP3. The highly conserved Y552 is involved in I3P binding, and in fact, Y552A is known to reduce the binding of this ligand [24]. The considerable difference in size due to the change in Y552C places the reactive sulfhydryl group of the cysteine too far from the ligand to participate in its binding (Figure 3C). Thus, Y552C may affect the binding of I3P and consequently, may interfere with calcium release to the cytoplasm. This finding may indicate that the mutated protein would not work properly, although this cannot be established with certainty.

To investigate PLEKHG2 p.T53I and *PNKD* c.-4C>G, luciferase analyses were performed. *PLEKHG2* is a RhoGTPase that catalyses the hydrolysis of guanosine triphosphate (GTP) to guanosine diphosphate (GDP), activated by heterotrimeric G protein βγ (Gβγ) subunits [25]. To assess whether the p.T53I mutation affects the activation of the PLEKHG2 downstream signalling pathway, we used a luciferase reporter assay in HEK293 cells under the control of the SRE promoter, whose transcription activity is known to be regulated by Rho GTPases. The transcriptional activation capacity expression of the mutated gene was significantly reduced by 25% compared to the control (Figure 3F). PNKD shares a high percentage of sequence identity (43%) with human hydroxyacylglutathione hydrolase (HAGH). The transcriptional activity of *PNKD* c.-4C>G resulted in a decrease to ~11% compared to the control (Figure 3F). For both variants, we detected altered luciferase activities. These in vitro findings do not guarantee with absolute certainty that the mutated protein has an abnormal activity. 

**Figure 4:**
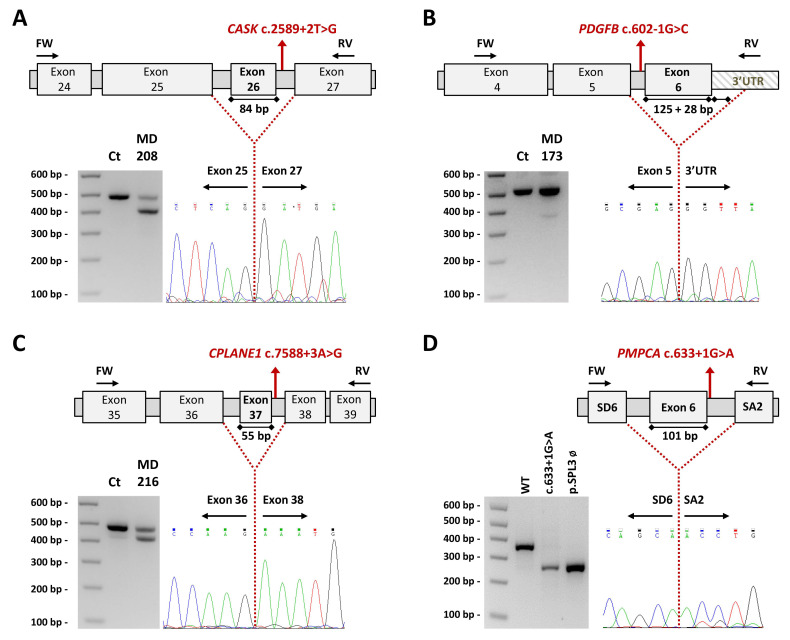
Impact on transcript processing of splicing mutations. Exons are indicated by boxes and introns are represented by horizontal bars (not to scale). Location of specific primers used in RT-PCR is indicated by arrows. Dotted lines represent the anomalous splicing outcome and sequences of the abnormal products are shown (electropherogram). Genomic mutations (in red) are harboured in a heterozygous state and the consensus transcript was also detected in patients’ samples further confirmed by specific band sequencing. (**A**–**C**) Reverse transcription-polymerase chain reaction (RT-PCR) products obtained from RNA of control and patients’ blood samples (agarose electrophoretic results) and schematic representation of the aberrant splicing events. (**A**) *CASK* c.2589+2T>G (MD-208) induces the complete skipping of exon 26, corresponding to the lower 402 bp band. (**B**) *PDGFB* c.602-1G>C (MD-173) causes the loss of the last exon of the gene plus 28 bp of the contiguous 3′ untranslated region (3′UTR) region presenting a 367 bp aberrant product absent in the healthy control. (**C**) *CPLANE1* c.7588+3G>A (MD-216) resulted in single exon 37 skipping leading to an alternative transcript 55 bp shorter than the expected. (**D**) *PMPCA* exon 6 minigene assay. Agarose gel shows the band pattern of transcripts obtained after overexpression of pSPL3-derived constructs: wild-type (WT) allele, mutant allele c.633+1G>A and empty vector ø. SD6 and SA2 are the resident exons of the pSPL3 reporting vector. In the absence of an inserted fragment, a product of 263 bp is obtained. The analysed variant causes the skipping of exon 6. bp, base pair; Ct, control; FW, forward primer; RV, reverse primer.

Four novel mutations, located on the consensus sequence at splice sites, causing the loss of at least one exon were detected (Figure 4): *CASK* c.2589+2T>G led to the skipping of exon 26 (84 bp); *PDGFB* c.602-1G>C to exon 6 plus part of the 3′UTR (untranslated region; 153 bp); *CPLANE1* c.7588+3A>G to exon 37 (55 bp); and *PMPCA* c.633+1G>A to exon 6 (101 bp). 

We performed immunofluorescence assays to determine the subcellular localization of NIPA1 p.P91R (Table 3), using p.G106R as pathogenic control associated with aberrant protein subcellular distribution [26]. The immunofluorescence assays performed with several cell markers (M6PR, GRP94, and EEA1) revealed partial colocalisation with these markers, although no differences were observed compared to WT (data not shown). Notwithstanding, the expression pattern using Na^+^, K^+^ ATPase showed that the mutated NIPA1 protein was concentrated in a perinuclear distribution, whereas the WT protein was distributed throughout the cytoplasm (Figure S3), suggesting an abnormal trafficking of mutated proteins as was established for the *NIPA1*-associated HSP phenotype [26,27]. 

## 3. Discussion

Our clinical series comprised 124 patients with MDs and/or CA. Genes related to NBIA disorders were initially studied in cases with brain iron deposits and/or a complex MDs phenotype that included ataxia, spasticity, and/or peripheral neuropathy. This first approach yielded a conclusive diagnosis for 59 patients. Most patients were PKAN (35.96%) or PLAN (12.28%), which represent the two major NBIA types [1]. Only four patients suffered from other NBIA disorders (mutations in *WDR45*, *FTL* or *CP*). Another PLAN subject (MD-341) who was directly studied by WES-proband, was later diagnosed. Strikingly, no mutations in the remaining NBIA genes were identified in subsequent analyses, which highlights that the abnormal accumulation of brain iron can be due to a primary mutation, but also to the neurodegenerative process that accompanies the disease. 

The haplotype for the *PANK2* locus in Roma patients confirmed that p.T528M was a founder mutation in Spain, just as other mutations have been previously reported for several Mendelian diseases [28,29,30]. The Roma population makes up around 1.5% of the total Spanish population, thus representing one of the largest Roma communities in the world (http://www.unioromani.org). PANK2 p.T528M likely represents an ancestral mutational event, since Roma PKAN patients from Spain and from Hungary shared the haplotype. The PANK2 p.T528M-associated haplotype was also constructed in the non-Roma Greek heterozygous patients, which may be the outcome of mixing with neighbouring populations. Ultimately, the different haplotypes obtained for the non-Roma Spanish proband heterozygous for PANK2 p.T528M showed that the mutation has arisen in different occasions throughout history. In fact, this has been reported in non-Roma PKAN-affected subjects [31,32,33,34]. PANK2 p.T528M seems to be relatively frequent, likely because homozygous individuals present with a milder clinical picture [35], which may have made its transmission easier through generations in the Roma population. 

MDs and ataxias are related to hundreds of genes with high phenotypic overlap. We developed a targeted method that tested between 498 genes in its first version [14] and 517 in its last version, as new genes of interest were reported. Our custom panel MovDisord displayed a capture with uniform coverage and high read depths in all samples, providing a cost-effective alternative, with a diagnosis success rate of 34.54% (19/55 patients). Nowadays, WES is probably the best strategy; WES-trio may yield ~40% of solved cases *versus* WES-proband with ~28% [36]. Nonetheless, we achieved a success rate of 70.0% (7/10 patients) and of 33.33% (3/9 patients) using WES-proband or WES-trio, respectively. It is worth mentioning that in the patients investigated by WES-trio, the most common genes were ruled out by the gene panel MovDisord. Altogether, we obtained a success rate of 52.63% (10/19 cases) using WES. In fact, WES-based strategies seem to display a diagnostic ceiling of ~50% [37], to a certain extent, due to the difficulty of interpreting new variants, hence the relevance of developing additional studies that help to understand their pathological nature. 

In the sixty-five patients investigated by panel MovDisord and/or WES, seventeen novel variants were identified, one of them a premature stop codon that was assumed to be detrimental. Together with the cases previously reported [12,13,14,15,16,17], we achieved experimental evidence of damage for 14 of these new genetic changes. Unfortunately, for some mutations, the possible *ad hoc* analyses were beyond our possibilities. The studies performed for *PNKD* c.-4C>G and NIPA1 p.P91R deserve a special mention. The luciferase assay revealed that *PNKD* c.-4C>G, detected in an affected man and in his asymptomatic daughter, may have a decreased transcriptional activity, supporting that the proper activity of the enzyme may be altered. Although we cannot categorically establish the causality of *PNKD* c.-4C>G, individuals carrying *PNKD* mutations who remain apparently unaffected have been reported, and in fact, incomplete penetrance seems to be relatively common in paroxysmal nonkinesigenic dyskinesia [38,39]. This phenomenon may explain the absence of clinical signs in the proband’s daughter, although it is also possible that she presents the first symptoms in her forties like her father. 

NIPA1 p.P91R was analysed by immunofluorescence and we concluded that intracellular trafficking would be impaired, as previously reported for other *NIPA1* mutations causing HSP [26,27]. NIPA1 p.P91R was detected in a patient with a complex phenotype, who also suffered from a partial trisomy 18p and a partial monosomy 18q. Known syndromes due to a similar chromosomal alteration have nothing to do with MDs [40,41]. A patient with a partial deletion of the chromosome 18q was reported with a complex phenotype, which shared some neurological findings with our proband: intellectual disability, sensorineural hearing loss and dystonia [42]. It is not possible to establish the contribution of NIPA1 p.P91R to the clinical outcome with the available data. 

In this cohort, 29 patients with phenotypes of MDs, spasticity, or ataxia were diagnosed with 23 distinct genetic conditions. Of those, five patients presented MDs such as dystonia or parkinsonism, spasticity, and suspicion of brain iron deposits or GP hypointensities; they were associated with variants in *FBXO7*, *FUCA1*, *GLB1*, *TPP1*, and *KIF1A*. In fact, isolated cases with GP hypointensities consistent with iron excess are known in at least 17 distinct genes [1], including *AP4M1* and *AP4S1*, which, like *FUCA1*, *GLB1*, and *TPP1,* are involved in lysosomal disorders. Lysosomes are implicated in the recycling of membranes, autophagy and even iron metabolism. It is tempting to speculate that abnormal iron accumulation may result from a defect in intralysosomal recycling. Patients with NBIA phenotype on MRI and mutations in *FUCA1* (1 case) or in *GLB1* (4 cases) have been previously described [43,44,45,46,47]. To our knowledge, our proband MD-153 is the first case with the early appearance of GP hypointensities and *TPP1* mutations. The three patients here described, MD-137 (*FUCA1*), MD-020 (*GLB1*), and MD-153 (*TPP1*), suffered from lysosomal diseases. Additionally, a subject with α-mannosidosis, also a lysosomal storage disease, who presented hypointensity signals in the GP was published [48]. Taken together, NBIA on MRI seems not to be so rare in some lysosomal disorders, especially in GM1-gangliosidosis, since so far, five unrelated cases are known (including our proband). Thus, *GLB1* has been suggested as an NBIA gene [49], although this depends on the authors. Single cases with NBIA phenotype have been reported for several genes such as *REPS1* or *CRAT* [50], and in occasions, they are described as NBIA genes [51], or as genes to be considered NBIA genes if additional cases are reported [52].

In 22 out of 29 solved patients, the phenotype was associated with CA, but only one was diagnosed with PLAN (MD-341). It is worth mentioning the early appearance of cerebellar cortical hyperintensities in seven patients with a non-progressive phenotype associated to variants in *ITPR1* (3), *SPTBN2* (2), *PMPCA*, and *KIF1A* genes, resembling the neuroimaging pattern recognised in neurodegenerative disorders such as PLAN, mitochondrial disorders, late onset GM2 gangliosidosis, and recently in our patient with *PRDX3*-associated neurodegeneration (PRAN) [17]. This paradox has already been described in other patients with ataxia and mutations in *KIF1A*, *ITPR1*, and *PMPCA* but also in *KCNC3*, and *CACNA1A* genes [53,54,55]. It has been presumed that the bright cortex in the cerebellum is a reactive gliosis to the neuronal cell loss and axonal swelling, but different hypotheses are pending to explain how diverse genetic disorders affecting predominantly the cerebellum early in life can present a neurodegenerative sign on neuroimaging, but stable or even improving neurodevelopment over the years. In addition, the three patients with *ITPR1* mutations also had a superior CA, a pattern very recently proposed to be related to *ITPR1* in 83% of a cohort that included carriers of the p.T267M and p.R269W variants like two of our patients [51].

Globally, our cohort presented a high phenotypic overlap as reported in MDs, ataxias, and NBIA disorders [1,2], with non-classical NBIA genes involved. Interestingly, two probands presented MDs, CA, and brain iron deposits or GP hypointensities, carrying mutations in *FBXO7* and *TPP1*, respectively. Spasticity and CA were a common combination in 10 patients with variants in *FBXO7*, *PLEKHG2*, *TPP1*, *ITPR1*, *KIF1A*, and *SPG7* genes; ataxia was also present in those with *ITPR1*, *KIF1A*, and *SPG7* mutations; two probands with mutations in *EXOSC3* suffered from early severe encephalopathy and CA, with progressive motor neuron signs after the first years of life.

## 4. Material and Methods

### 4.1. Patients

The study was conducted in accordance with the Declaration of Helsinki, and was approved by the Ethic Committees at Hospital Sant Joan de Déu in Barcelona (protocol code PIC-27-15 dated 12 March 2015) and Hospital Universitari i Politècnic La Fe in València (protocol codes: 2019/0052 dated 22 May 2019, and 2021/65/1 dated 22 September 2021). 

Our cohort comprised 124 paediatric and adult patients with MDs and/or CA. PLAN and PKAN cohorts were previously described [5,35]. Acquired aetiologies were fully excluded in all of them. Demographic and familial history was systematically collected. Phenotyping included standardised assessments of ataxia, MDs, and other neurological symptoms such as spasticity, muscle weakness, or signs of peripheral neuropathy. Based on the phenotypic traits, we classified the patients according to two clinical presentations: (1) MDs such as dystonia, parkinsonism or tremor; spasticity; with or without brain iron deposits; (2) Early-onset ataxia or encephalopathy and CA, with or without brain iron deposits.

### 4.2. Neuroimaging Approaches

Brain MRI studies were performed in 1.5- and 3-T scanners, depending on the referral hospitals. The identification of iron deposit signs in basal ganglia, particularly in *globus pallidus* (GP) and *substantia nigra* (SN), was ascertained by iron-sensitive sequences, especially susceptibility weighted imaging (SWI) and T2-weighted imaging (T2WI). In MRI studies without these specific sequences, T2-weighted (T2W) hypointensities in basal ganglia were considered only suspicious with regard to iron deposits, according to normal age-dependent signals [3]. CA was ascertained by loss of cerebellar volume in at least two consecutive cerebral MRI studies. To assess the progression of CA, a quantitative analysis by mid-sagittal vermis relative diameter (MVRD) was performed in cerebral MRIs [5,13]. Additionally, cerebellar cortical hyperintensities were ascertained on T2-weighted or FLAIR MRI sequences, since they are associated with neurodegenerative phenotypes as PLAN [5].

### 4.3. Genetic and Genomic Studies

#### 4.3.1. Genetic Analysis of NBIA Genes

In the first phase, the study was conducted based on the patients’ clinical findings. In 114 patients, the main NBIA genes, *PANK2*, *PLA2G6*, *WDR45*, *FTL*, and *CP*, were screened by Sanger sequencing as previously described [5,35], including the analysis of large deletions and/or duplications in *PANK2* and *PLA2G6* by Multiplex Ligation-dependent Probe Amplification (SALSA MLPA probemix P120, MRC Holland, Amsterdam, The Netherlands) (Figure 1). 

To investigate if the *PANK2* c.1583C>T (rs137852967) detected in homozygosis in Roma patients was a founder mutation, we constructed the haplotype for the *PANK2* locus. We included eight homozygous Roma probands from Spain (7) and from Hungary (1), and four heterozygous non-Roma probands from Greece (3) and from Spain (1). We selected five extragenic markers (3 microsatellites and 2 SNPs) and three intragenic SNPs from the UCSC Genome Browser (https://genome.ucsc.edu/, accessed on 4 November 2021), the GeneLoc (https://genecards.weizmann.ac.il/geneloc/index.shtml, accessed on 4 November 2021) or the dbSNP (https://www.ncbi.nlm.nih.gov/snp/, accessed on 4 November 2021), which spanned a 1.06 Mb region flanking the *PANK2* gene: cen_rs1078152-*D20S867*-rs6084513-rs137852967-rs6037695-rs7270329-*D20S889*-*D20S116*-rs1628323_tel. Protocol has been described previously [56]. Forward PCR primers (available upon request) were labelled with FAM fluorescent dyes (Sigma Aldrich, St. Louis, MO, USA). Amplicons were electrophoresed using an ABI3730xl (Applied Biosystems, Foster City, CA, USA) and analysed with the Peak Scanner Software v2.0, at the Service of Genomics and Translational Genetics (CIPF, Valencia, Spain). 

#### 4.3.2. Gene Panel, WES and Chromosomal Microarray Analysis

The 55 remaining patients without a conclusive result after the analysis of the NBIA genes were investigated using a custom panel MovDisord (Figure 1). The Table S1 shows the 517 genes involved in MDs and ataxia included in the last version of the panel MovDisord, which was reported in its first version [14].

Later, nine remaining patients without genetic diagnoses were investigated by WES-trio, and to compare panel genes *versus* WES, 10 additional patients were directly investigated by WES-proband as previously reported [17], except for patient MD-277, which was investigated as previously reported [12].

Bioinformatics analyses (filtering data, study of the novelty of the candidate variants and CNVs, copy number variants) were carried out as previously described [17]. WES data from unsolved patients were recently reanalysed using the platform RD Connect Genome-Phenome Analysis Platform (https://platform.rd-connect.eu/, accessed on 4 November 2021).

Molecular karyotype was performed using genomic DNA from peripheral blood leukocytes and the CytoScan 750 K Array platform (Affymetrix Inc., Santa Clara, CA, USA) according to the manufacturer’s protocol. Hybridised arrays were scanned on an Affymetrix GeneChip Scanner 3000 and the resulting files were analysed with Chromosome Analysis Suite (ChAS) (Affymetrix Inc., Santa Clara, CA, USA) software v3.1, based on the reference genome sequence of the UCSC Genome Browser hg19. DGV (http://dgv.tcag.ca/dgv/app/home, accessed on 16 July 2021) and DECIPHER (https://decipher.sanger.ac.uk/, accessed on 16 July 2021) were used for the interpretation of CNVs. 

### 4.4. Studies to Investigate the Pathogenicity of Novel Variants

#### 4.4.1. Structural Modelling: ITPR1 and FUCA1

Figure 3 was prepared using *PYMOL* Molecular Graphics System, v2.0 Schrödinger, LLC (https://pymol.org, accessed on 15 September 2021). ITPR1 p.Y552C was modelled in the crystal structure of the IP3 binding domain of ITPR1 (residues 236-602, PDB entry 1N4K) [24]. FUCA1 p.R47P was modelled by SWISS-MODEL [57], from the crystal structure of TmFUCA1 (PDB entries 1HL8, 1HL9 and 1ODU) [21].

#### 4.4.2. Luciferase Reporter Assays: PLEKHG2 and PNKD

The reporter vector pGL4.33[luc2P/SRE/Hygro] (Promega, Madison, WI, USA) and *PLEKHG2* (NM_022835.3) cloned into the pcDNA3.1:myc (N-ter) vector, kindly ceded by Dr. Chen Songhai [25], were employed in *PLEKHG2* assays. For *PNKD*, a fragment of 933 bp of the 5’ upstream sequence of the gene was amplified by PCR from a healthy control using the Herculase II Fusion DNA Polymerase (Agilent Technologies, Foster City, CA, USA) and cloned into the pGL4.24[luc2P/minP] reporter vector (Promega, Madison, WI, USA) between *Xho*I/*Hind*III restriction sites. The mutants (PLEKHG2 p.T53I and *PNKD* c.-4C>G) were obtained using the aforementioned technique. Primers are available upon request. HEK293T cells were cultured with complete DMEM (supplemented with 10% heat-inactivated FBS, 5 g/L D-glucose, 1% P/S and 1% L-glutamine) in 24-well plates. The following day, cells were transiently transfected using FuGENE HD Transfection Reagent (Promega, Madison, WI, USA) with the pertinent pGL4 constructs, and renilla luciferase normalisation vector (pRL-TK) with different p.GL4: pRL-TK ratio for each experiment, being 30:1 for *PNKD* and 3:1 for *PLEKHG2*. Furthermore, in the case of *PLEKHG2*, the pGL4.33[luc2P/SRE/Hygro] vector was co-transfected with wild type or mutated PLEKHG2 and after six hours the cell medium was changed to complete DMEM without FBS, as previously described [25,58]. For both assays, after 24 h, luciferase activity was assessed using the Dual-Luciferase^®^ Reporter Assay System (Promega, Madison, WI, USA). Three independent experiments were carried out for statistical analysis by unpaired two-sample *t* test.

#### 4.4.3. Transcript Analysis and Minigenes: CASK, PDGFB, CPLANE1/C5orf42 and PMPCA

Protocols for both approaches are described in Sánchez-Monteagudo et al. [59]. Primers are available upon request.

#### 4.4.4. NIPA1 Subcellular Location

The NIPA1 (NM_144599.4) tagged with FLAG in C-ter and cloned in pcDNA3.1 vector was acquired from GenEZ™ ORF cDNA Clones (GenScript, Piscataway, NJ, USA). The NIPA1 p.P91R and p.G106R missense mutations were introduced using a Site-Directed Mutagenesis kit (Agilent, Santa Clara, CA, USA) using specific primers for each mutation (available upon request). HeLa cells were grown in complete DMEM and transiently transfected with 2 µg of NIPA1 wild-type (WT), p.P91R, or p.G106R with FuGENE HD Transfection Reagent (Promega, Madison, WI, USA). After 24 h, the cells were fixed, permeabilised and blocked as previously described [13]. The samples were incubated with the primary antibodies anti-FLAG (Sigma-Aldrich, Saint Louis, MO, USA) and anti-Na^+^/K^+^-ATPase, anti-mannose-6-phosphate receptor (M6PR), anti-glucose-regulated protein 94 KDa (GRP94), or anti-early endosome antigen 1 (EEA1; Abcam, Cambridge, UK). The following day, they were exposed to the appropriate secondary antibodies conjugated with fluorophores (Invitrogen, Carlsbad, CA, USA) and examined using the SP2-Leica confocal microscope (Leica, Wetzlar, Germany).

## 5. Conclusions

The NGS technology has made it possible to accelerate the diagnosis of Mendelian MDs, but the number of unsolved cases is still too high. The interpretation of the genetic variants and their phenotypic consequences is a challenge. The low prevalence of each clinical form, the great genetic heterogeneity, and the notable variable expressivity complicate the diagnosis. This report adds new genetic and clinical pieces to the puzzle of MDs and/or ataxias, and contributes to improving the genetic counselling and care of these patients with rare diseases. We have to work hard in order to “enable all people living with a rare disease to receive an accurate diagnosis, care and available therapy within one year of coming to medical attention” [60]. 

## Figures and Tables

**Table 1:** Genetic findings of the 29 solved cases.

Patient	Gene	RefSeq	Position	DNA Change	Protein Change	Prediction ^◊^	rs number (MAF) ^ǂ^	References PMID ^§^	Method
MD-208	*CASK*	NM_003688.3	X:41383202	c.2589+2T>G	―	P	NA	None	Gene Panel
MD-216	*CPLANE1*	NM_023073.3	5:37201821	c.3379T>G	p.S1127A	LP	rs776423792 (1.193 × 10^−5^)	None	Gene Panel
			5:37164372	c.7588+3A>G	―	P	NA	None	
MD-122	*EXOSC3*	NM_013042.3	9:37783990	c.395A>C	p.D132A	P	rs141138948 (4.067 × 10^−4^)	22544365, 23564332, 23564332, 23975261, 24524299, 25533962, 27777260, 28687512, 30950035, 31692161	Gene Panel
				HOMOZYGOSIS (No Consanguinity)					
MD-012	*EXOSC3*	NM_013042.3	9:37783990	c.395A>C	p.D132A	P	rs141138948 (4.067 × 10^−4^)	22544365, 23564332, 23564332, 23975261, 24524299, 25533962, 27777260, 28687512, 30950035, 31692161	Gene Panel
				HOMOZYGOSIS (No Consanguinity)					
MD-018 ^#^	*FBXO7*	NM_012179.3	22:32875213	c.368C>G	p.S123 *	P	NA	32767480	Gene Panel
				HOMOZYGOSIS (Consanguinity)					
MD-137	*FUCA1*	NM_000147.4	1:24194637	c.140G>C	p.R47P	LP	NA	None	Gene Panel
				HOMOZYGOSIS (Consanguinity)					
MD-020	*GLB1*	NM_000404.3	3:33114105	c.176G>A	p.R59H	P	rs72555392 (3.607 × 10^−5^)	10338095, 17664528, 28939701, 31761138	Gene Panel
			3:33114174	c.107A>G	p.Y36C	P	rs748345527 (4.809 × 10^−5^)	None	
MD-270	*HEXA*	NM_000520.5	15:72646027	c.459+5G>A	―	P	rs762060470 (2.829 × 10^−5^)	1837283, 25525159	Gene Panel
			15:72638893	c.1305C>T	―	P	rs587779406 (8.488 × 10^−5^)	20363167, 25606403	
MD-320	*ITPR1*	NM_00116872.1	3:4687357	c.800C>T	p.T267M	P	rs797044955 (NA)	24091540, 28659154, 29878067, 29925855, 29925855, 30842224, 31632679, 32695065	WES-proband
MD-106	*ITPR1*	NM_00116872.1	3:4687362	c.805C>T	p.R269W	P	NA	27062503, 25533962, 28826917, 28191890, 28135719, 28659154, 29925855	Gene Panel
MD-041	*ITPR1*	NM_00116872.1	3:4706967	c.1655A>G	p.Y552C	LP	NA	None	Gene Panel
MD-200	*KIF1A*	NM_001244008.1	2:241725854	c.506C>G	p.R169T	LP	NA	34121983	Gene Panel
MD-189	*KIF1A*	NM_001244008.1	2:241715280	c.946C>T	p.R316W	P	NA	25265257, 26354034, 28554332	Gene Panel
MD-299	*KIF1A*	NM_001244008.1	2:241715280	c.946C>T	p.R316W	P	NA	25265257, 26354034, 28554332	WES-proband
MD-319	*LRRK2*	NM_198578.4	12:40734202	c.6055G>A	p.G2019S	P	rs34637584 (4.884 × 10^−4^)	15726496, 16102999, 16333314, 16750377, 16966501, 17060589, 17116211, 17210620, 17200152, 19072560, 20008657, 19283415, 21686713, 19302196, 19741132 σ	WES-proband
				HOMOZYGOSIS (Consanguinity)					
MD-277 ^#^	*NR4A2*	NM_006186.4	2:157184954	c.956G>A	p.R319Q	LP	NA	33585677	WES-trio
MD-173	*PDGFB*	NM_002608.3	22:39621853	c.602-1G>C	―	P	NA	None	Gene Panel
MD-252	*PNKD*	NM_015488.4	2:219187987	c.-4C>G	―	LP	rs1461115674 (8.152 × 10^−6^)	None	Gene Panel
MD-341	*PLA2G6*	NM_003560	22:38519251	c.1442T>A	p.L481Q	LP	rs587784330 (5.922 × 10^−6^)	16783378, 24870368, 25164370	WES-proband
				HOMOZYGOSIS (Consanguinity)					
MD-181	*PLEKHG2*	NM_022835.2	19:39905680	c.158C>T	p.T53I	P	NA	None	WES-trio
				HOMOZYGOSIS (Consanguinity)					
MD-323	*PMPCA*	NM_015160.2	9:139310844	c.633+1G>A	---	P	rs1442110087 (7.961 × 10^−6^)	None	WES-proband
			9:139306513	c.136T>C	p.S46P	LP	NA	None	
MD-174 ^#^	*PRDX3*	NM_006793.5	10:120931956	c.489C>G	p.R163E	P	NA	35766882	WES-trio
				HOMOZYGOSIS (Consanguinity)					
MD-296	*QARS1*	NM_005051	3:49138870	c.794G>A	p.R265H	LP	rs916890735 (6.575 × 10^−6^)	None	WES-proband
				HOMOZYGOSIS (Consanguinity)					
MD-126 ^#^	*REEP1*	arr[hg19]2p11.2 (chr2:83,335,425-87,271,924; Hg 19)				P	―	22062632, 24986827	Gene Panel
MD-307	*RPGRIP1L*	NM_015272	16:53720424	c.697A>T	p.K233*	P	rs121918197 (2.788 × 10^−5^)	17558409, 25525159	WES-proband
			16:53686828	c.1769_1770delCT	p.S590Cfs*	P	NA	None	
MD-159 ^#^	*SPG7*	NM_003119.3	16:89613145	c.1529C>T	p.A510V	LP	rs61755320 (2.899 × 10^−5^)	18799786, 20981092, 20186691, 21623769, 22995991, 23269439, 22571692, 25133958, 25525159, 26626314, 29057857, 27957547, 29026558, 28362824, 28832565, 29482223, 29913018, 30369941, 31433872, 30098094, 31316545, 29915382, 30537300, 31692161	Gene Panel
			16:89616953	c.1715C>T	p.A572V	P	rs72547551 (3.537 × 10^−5^)	14985266, 25681447, 29482223	
MD-219 ^#^	*SPTBN2*	NM_006946.3	11:66483417	c.193A>G	p.K65E	P	NA	33801522	Gene Panel
MD-207 ^#^	*SPTBN2*	NM_006946.3	11:66481110	c.764A>G	p.D255G	P	NA	33801522	Gene Panel
MD-153	*TPP1*	NM_000391.3	11:6636487	c.1340G>A	p.R447H	P	rs119455956 (7.953 × 10^−5^)	10330339, 19038966, 20340139, 26143525, 29655203	Gene Panel
			11:6640007	c.229G>C	p.G77R	P	rs121908195 (7.969 × 10^−5^)	26633542, 31741823	

AD, Autosomal Dominant; AR, Autosomal Recessive; LP, Likely Pathogenic; NA, Not-Available; P, Pathogenic; PMID, PubMed Identifier; RefSeq, Reference Sequence; SCA, Spinocerebellar Ataxia; SPG: Spastic Paraplegia; WES, Whole Exome Sequencing; XLD, X-linked dominant. ^◊^ Prediction of pathogenicity performed according to the ACMG/AMP guidelines [11]. ^ǂ^ Consulted database gnomAD v2.1.1, except for rs916890735 that is only annotated in gnomAD v3.1.1 (accessed on 1 August 2022). ^§^ References associated with each mutation in the Human Mutation Database (HGMD^®^) Professional 2022.2 (Qiagen, Santa Clarita, CA, USA; accessed om 1 August 2022). σ The LRRK2 p.G2019S change is reported in at least 119 articles. ^#^ Clinical features of patients MD-018, MD-277, MD-174, MD-126, MD-159, MD-219, and MD-207 were previously reported [12,13,14,15,16,17].

**Table 2:** Clinical features of the 29 solved cases.

Patient Sex	Gene	Origin	Presentation Inheritance	Disease (OMIM)	Age of Onset Age at Testing	Early Symptoms	Brain MRI	Additional Clinical Features
MD-208 Female	*CASK*	Spain	de novo XLD	Mental retardation and microcephaly with pontine and cerebellar hypoplasia (300749)	1 mo 3 yo	Hypotonia Microcephaly DD	Severe PCH	Mild limb dystonia, choreoathetosis, progressive scoliosis from 3 yo
MD-216 Male	*CPLANE1*	Morocco	Familial AR	Joubert syndrome 17 (614615)	1 yo 10 yo	DD Hypotonia, ataxia OMA	Molar tooth sign Cerebellar hypoplasia	ID, ataxia and OMA improve with age
MD-122 Female	*EXOSC3*	Spain	Sporadic AR	Pontocerebellar hypoplasia type 1B (614678)	3 mo 3 yo	Hypotonia, axial hyperextension, strabismus	Progressive vermis atrophy	Spasticity progressing to flaccid paralysis from 3-yo, g-tube feeding, neurogenic EMG pattern, *exitus* at 4 yo
MD-012 Male	*EXOSC3*	Spain	Sporadic AR		4 mo 8 yo	Hypotonia, DD	Progressive vermis atrophy	Spasticity from 4 yo, axonal neuropathy with loss of tendon reflexes from 5 yo, g-tube feeding, limb dystonia from 6 yo
MD-018 ^#^ Female	*FBXO7*	Morocco	Sporadic AR	Parkinson disease 15 (260300)	2 yo 21 yo	Mild DD, infection-triggered acute ataxia at 2 yo	CA from 10 yo Brain iron deposits from 15 yo	Absence epilepsy from 5 yo, acute encephalopathy at 12 yo, progressive deterioration, spastic paraparesis, drug-resistant seizures, optic neuropathy, parkinsonism from 15 yo
MD-137 Male	*FUCA1*	Greece	Sporadic AR	Fucosidosis (230000)	3 yo 10 yo	Speech difficulties, gait disturbances	GP T2 hypointensity from 10 yo	Static encephalopathy, progression of gait disorder with dystonia, ID, dysmorphic features, ADHD symptoms, motor stereotypes
MD-020 Female	*GLB1*	Spain	Sporadic AR	GM1-gangliosidosis (230500, 230600, 230650)	3 yo 12 yo	Speech difficulties, motor deterioration	Brain iron deposits from 10 yo	Oromandibular dystonia, dysarthria, neuropathy, spasticity, scoliosis
MD-270 Female	*HEXA*	Spain	NA AR	GM2-gangliosidosis (272800)	26 yo 23 yo	DD, hypotonia	CA	Cerebellar syndrome, stereotyped behaviour, progressive cognitive decline
MD-320 Male	*ITPR1*	Senegal	de novo AD	SCA15 (606658) SCA29 congenital nonprogressive (117360)	3 mo 5 yo	DD, hypotonia, gaze-evoked nystagmus	CA Cerebellar cortical hyperintensity from 3 yo (upper > lower hemispheres)	Ataxia, NPCA, strabismus, ID, mild lower limbs spasticity
MD-106 Male	*ITPR1*	Morocco	de novo AD		<1 yo 6 yo	DD, hypotonia	CA Cerebellar cortical hyperintensity from 3 yo (upper > lower hemispheres)	Ataxia, NPCA, strabismus, gaze-evoked nystagmus, ID, lower limbs spasticity
MD-041 Male	*ITPR1*	Spain	de novo AD		2 mo 3 yo	Gaze-evoked nystagmus, hypotonia	CA Cerebellar cortical FLAIR hyperintensity at age of 4 (upper > lower hemispheres)	Ataxia, NPCA, strabismus, abnormal ocular movements, ID
MD-200 Male	*KIF1A*	Spain	de novo AD	NESCAV syndrome (614255)	<1 yo 12 yo	DD, microcephaly, nystagmus	CA	Ataxia, NPCA, lower limbs spasticity, seizures (4 yo), dysphagia, intellectual disability, axonal neuropathy, optic atrophy (9 yo)
MD-189 Male	*KIF1A*	Portugal	de novo AD	SPG30 (610357)	12 mo 22 yo	DD, visual deficit	CA Brain iron deposits from 17 yo	Ataxia, NPCA, optic atrophy, stuttering (2 yo), lower limbs spasticity (3 yo), cognitive regression (4 yo), peripheral neuropathy, seizures (14 yo)
MD-299 Male	*KIF1A*	Spain	de novo AD	SPG30 (610357)	11 mo 9 yo	DD, hypotonia	CA Cerebellar cortical and deep WM FLAIR hyperintensity	Ataxia (2 yo), NPCA, acquired microcephaly, lower limbs spasticity (3 yo), cognitive impairment, no regression
MD-319 Male	*LRRK2*	Morocco	Sporadic AR	Parkinson disease (607060)	5 yo 14 yo	Tremor	Normal	Hands tremor, cervical tremor, mild bradykinesia, hoarseness voice (13 yo)
MD-277 ^#^ Male	*NR4A2*	Spain	de novo AD	AD early-onset dystonia-parkinsonism with intellectual disability (601828)	12 yo 29 yo	Mild DD from 1 yo	Normal	Borderline IQ (77; 7 yo), motor tics from 16-yo, dystonia and parkinsonism from 28 yo
MD-173 Female	*PDGFB*	Spain	Familial AD	Basal ganglia calcification idiopathic 5 (615483)	19 yo 19 yo	Postural and intentional tremor	Calcifications	Tremor
MD-252 Male	*PNKD*	Spain	Familial? AD	Paroxysmal nonkinesigenic dyskinesia 1 (118800)	49 yo 49 yo	Paroxysmal dystonia	Normal	Dystonia, parkinsonism
MD-341 Female	*PLA2G6*	Morocco	Sporadic AR	Infantile neuroaxonal dystrophy 1 (256600) NBIA2B (610217) Parkinson disease 14 (612953)	12 mo 5 yo	Ataxic gait, DD	CA Cerebellar cortical FLAIR hyperintensity	Ataxia, dysmetria, hypotonia, spasticity, axonal motor impairment, optic atrophy, intellectual disability
MD-181 Male	*PLEKHG2*	Spain	Sporadic AR	Leukodystrophy and acquired microcephaly with or without dystonia (616763)	1 mo 6 yo	Hypotonia, DD	Thalamic lesions CA	Spastic-dystonic tetraparesia from 6 mo, bilateral cataracts, OMA, neuropathy
MD-323 Male	*PMPCA*	Spain	Sporadic AR	SCAR2 (213200)	21 mo 9 yo	Motor development delay, ataxia, nystagmus	CA Cerebellar cortical hyperintensity	Ataxia, NPCA, mild cognitive impairment
MD-174 ^#^ Male	*PRDX3*	Morocco	Sporadic AR	NA	2 yo 3 yo	Non-triggered acute cerebellar syndrome	Rapid progression of CA	Ataxia, chronic cerebellar syndrome following acute onset, demyelinating neuropathy from 5 yo
MD-296 Male	*QARS1*	Morocco	Sporadic AR	Microcephaly, progressive seizures, and cerebral and cerebellar atrophy (615760)	18 mo 13 yo	Febrile seizures, DD, atypical absences	CA	ID, language impairment, drug-resistant epilepsy with non-motor and motor seizures
MD-126 ^#^ Female	*REEP1*	Spain	de novo AD	SPG31 (610250)	33 yo 35 yo	Parkinsonism	Calcifications	Parkinsonism, tremor, spasticity, dystonia, slow saccades, dysarthria
MD-307 Male	*RPGRIP1L*	Spain	Sporadic AR	COACH syndrome (216360) Joubert syndrome 7 (611560) Meckel syndrome 5 (611561)	1 mo 1 yo	Hypotonia, nystagmus, strabismus	PCH	Profound DD, g-tube feeding, awake apneas, seizures, dyskinesia, *exitus* at 24 mo
MD-159 ^#^ Male	*SPG7*	Spain	Sporadic AR	SPG7 (607259)	17 yo 26 yo	Dystonia, postural instability, dysarthria	CA	Mild dystonic gait with mild spastic-ataxia gait, dysmetric saccades, gaze-evoked nystagmus, intention tremor
MD-219 ^#^ Male	*SPTBN2*	Spain	de novo AD	SCA5 (600224)	4 mo 11 yo	Hypotonia, transient upgaze deviation	Severe CA Cerebellar cortical hyperintensity	Ataxia, NPCA, moderate ID
MD-207 ^#^ Male	*SPTBN2*	Spain	de novo AD	SCA5 (600224)	12 mo 8 yo	Motor delay, DD	Severe CA Cerebellar cortical hyperintensity	Ataxia, NPCA, ADHD, borderline IQ
MD-153 Male	*TPP1*	Cuba	Sporadic AR	Ceroid lipofuscinosis neuronal 2 (204500) SCA7 (609270)	5 yo 13 yo	Speech delay, stuttering, motor clumsiness	GP FLAIR low signal, cerebral and CA, white matter T2-high signal	Limb and orofacial dystonia from 11 yo, spasticity, cognitive decline, no seizures

AD, Autosomal Dominant; ADHD, Attention-Deficit Hyperactivity Disorder; AR, Autosomal Recessive; CA, Cerebellar Atrophy established in consecutive neuroimaging; COACH, Cerebellar vermis hypo/aplasia, Oligophrenia, Congenital ataxia, Ocular coloboma, and Hepatic fibrosis; FLAIR, fluid attenuated inversion recovery; GP, Globus pallidus; DD, Developmental Delay; EMG, Electromyogram; ID, Intellectual Disability; IQ, Intelligence Quotient; mo, months old; MRI, Magnetic Resonance Imaging; NA, Not-available; NBIA, Neurodegeneration with Brain Iron Accumulation; NESCAV, Neurodegeneration and Spasticity with or without Cerebellar Atrophy or Cortical Visual impairment; NPCA, Non-Progressive Congenital Ataxia; OMA, Oculomotor Apraxia; OMIM, Online Mendelian Inheritance in Men; PCH, Ponto-Cerebellar Hypoplasia; SCA, Spinocerebellar Ataxia; SCAR, SCA Autosomal Recessive; SPG, Spastic Paraplegia; WM, White Matter; XLD, X-Linked Dominant; yo, years old. ^#^ Clinical features of patients MD-018, MD-277, MD-174, MD-126, MD-159, MD-219 and MD-207 were previously reported [12,13,14,15,16,17].

**Table 3:** Genetics and main clinical features of controversial cases.

Patient Sex	Gene	Candidate Mutation rs Number (MAF) ^§^	Onset Status Inheritance	Disease (OMIM) Inheritance	Main Clinical Features	References PMID ^§^	Observations
MD-168 Male	*NIPA1*	arr[hg19] 15q11.2(22,770,421–23,277,436)×1 507 Kb deletion that includes seven genes: *TUBGCP5, CYFIP1, NIPA2, NIPA1, LOC283683, WHAMMP3, GOLGA8I*	2 yo Sporadic case Heterozygosis AD	The 15q11.2 BP1-BP2 microdeletion syndrome (615656) AD	Autism, obsessive phobic disorder, language delay, dysarthria, severe hypermetropia, generalised dystonia	31451536, 25689425, 30909440, 30542208, 19328872, 21359847, 28387067, 32117010	The 15q11.2 BP1-BP2, although associated with neurodevelopmental disorders in multiple papers, should not be considered as a disease causing mutation, since this microdeletion or reported microduplication may explain only a small fraction of the clinical phenotype
MD-143 Male	*NIPA1*	arr[hg19] 15q11.2(22,770,421–23,214,655)×1 444 Kb deletion that includes six genes: *TUBGCP5, CYFIP1, NIPA2, NIPA1, LOC283683, WHAMMP3*	73 yo Sporadic case Heterozygosis AD		Chorea, parkinsonism		
MD-232 Male	*NIPA1*	c.272G>C (p.P91R) novel	12 yo Sporadic case Heterozygosis AD	Spastic paraplegia 6 (600363) AD	GP T2 hypointensity, lower limb spasticity-dystonia, intellectual disability, sensorineural deafness	novel	The proband also had a 18p duplication and a 18q deletion with unclear clinical implications.
MD-179 Female	*GCH1*	c.671A>G (p.K224R) rs41298442(MAF: 3.709 × 10^−4^)	20 yo Sporadic case Heterozygosis AD	Dystonia DOPA-responsive (128230) AD	GP T2 hypointensity, dystonia, dysarthria, upper limbs tremor	8852666, 12391354, 15303002, 25497597, 30314816	Her two asymptomatic children carried the mutation in heterozygosis
MD-342 Female	*PARK2* *PRKN*	c.220_221dupTG (p.W74Cfs*) rs746646126(MAF: 3.191 × 10^−5^)	9 yo Sporadic case Homozygosis AR	Parkinson disease juvenile type 2 (600116) AR	Ataxia, motor delay, intention tremor, dysarthria, hypotonia, spasticity	10072423	The proband’s phenotype does not fit with the *PARK2*-associated parkinsonism
MD-347 Female	*SACS*	c.4016C>A (p.Q1143K) rs144267558(MAF: 1.066 × 10^−3^)	2 mo Sporadic case Compound Heterozygosis AR	ARSACS (270550) AR	Cerebellar vermis hypoplasia, oculomotor apraxia, improving with age	29915382	MD-347 seems to be a carrier of a deleterious mutation (p.Q1143K), whereas p.N4573H (VUS) likely does not contribute to the clinical outcome.
		c.14306A>C (p.N4573H) rs34382952(MAF: 3.124 × 10^−3^)				None	

AD, Autosomal Dominant; AR, Autosomal Recessive; ARSACS, Autosomal Recessive Spastic Ataxia type Charlevoix Saguenay; GP: Globus pallidus; MAF, Minor Allele Frequency; NBIA, Neurodegeneration with Brain Iron Accumulation; mo, months old; OMIM: Online Mendelian Inheritance in Men; PMID, PubMed Identifier; yo, years old; VUS: Variant of Uncertain Significance; XLD: X-Linked Dominant. Consulted database gnomAD v2.1.1. ^§^ References associated with each mutation in the Human Mutation Database (HGMD^®^) Professional 2022.2 (Qiagen, Santa Clarita, CA, USA; accessed on 1 August 2022).

## Data Availability

All data relevant to the study are included in the article or uploaded as supplementary information.

## Supplementary Materials

The following supporting information can be downloaded at: https://www.mdpi.com/article/10.3390/ijms231911847/s1.
