# Supplementary material for: Mutations, Genes, and Phenotypes Related to Movement Disorders and Ataxias
Source: Int J Mol Sci. 2022 Oct 6;23(19):11847. doi: 10.3390/ijms231911847 (PMC9570320; doi:10.3390/ijms231911847)
Supplement: Supplementary file 1 [file ijms-23-11847-s001.zip › ijms-1927397-supplementary.pdf]

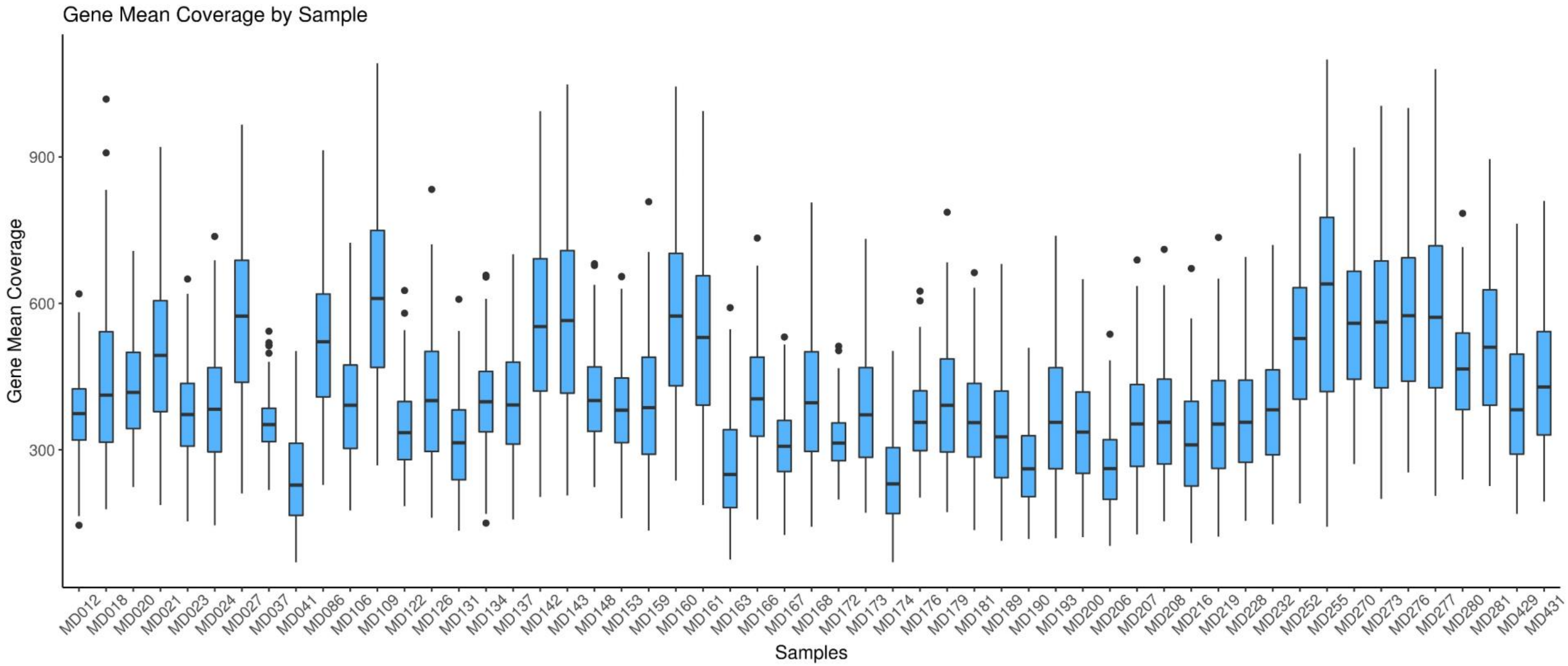

Supplementary Figure S1. Depth of coverage of each of the 55 samples analysed using the custom gene panel MovDisord.

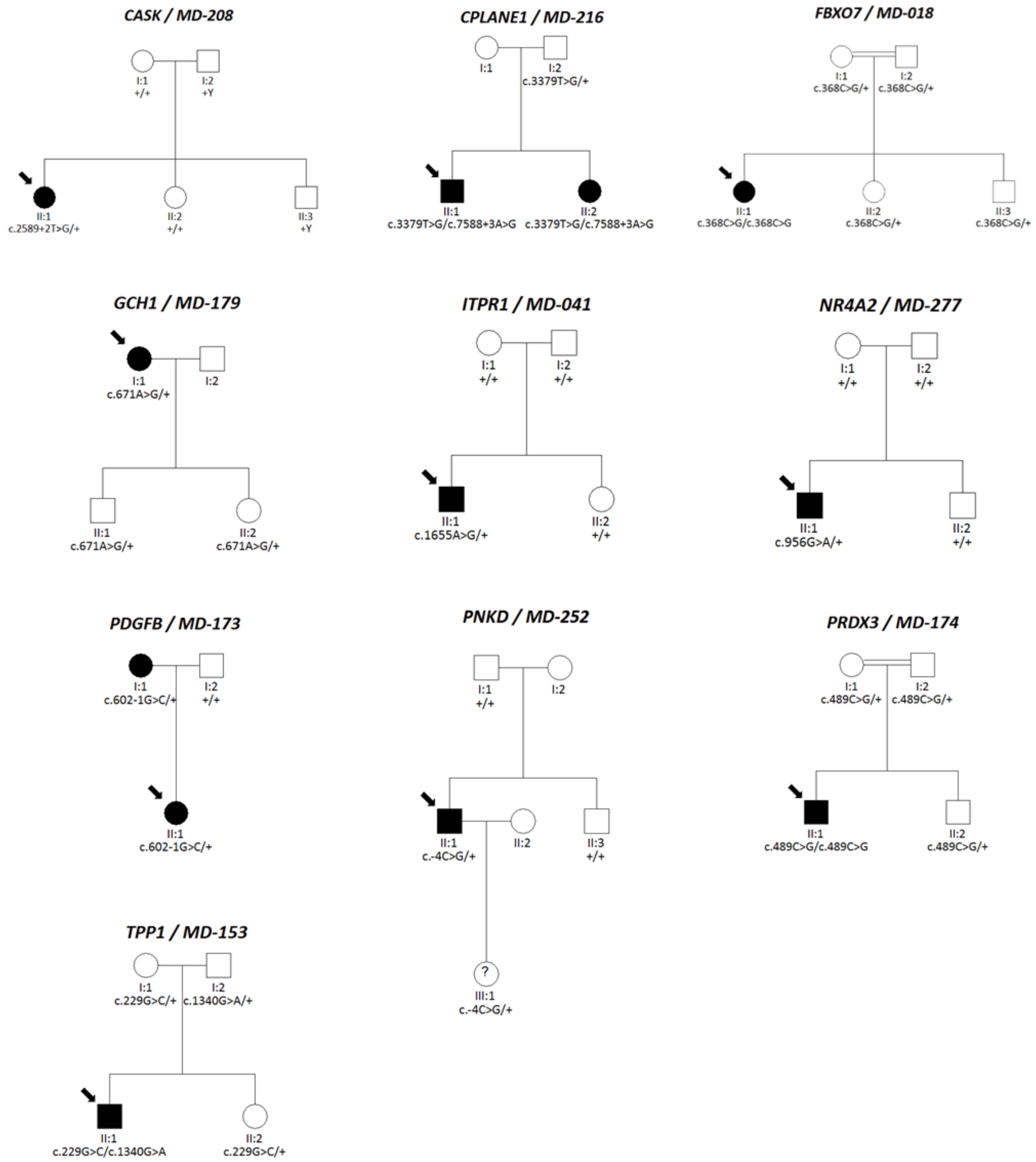

Supplementary Figure S2. Segregation analysis of disease-causing mutations. Only families for which DNA was available from parents and other relatives are included. At the top of each pedigree, the responsible gene and the proband's code are indicated. The arrow points out the proband. Genotypes for each deleterious variant are shown below each symbol/subject; those individuals without genotype were not investigated because DNA was not available. Plus (+) symbol refers to the absence of the causative changes. A question mark (?) on the MD-252's daughter (*PNKD*) means that this girl (25 years old) is currently asymptomatic. All the implicated genes are located in autosomes, except *CASK* that is located on chromosome X. MD-018 (*FBXO7*) and MD-174 (*PRDX3*) were born to consanguineous parents.

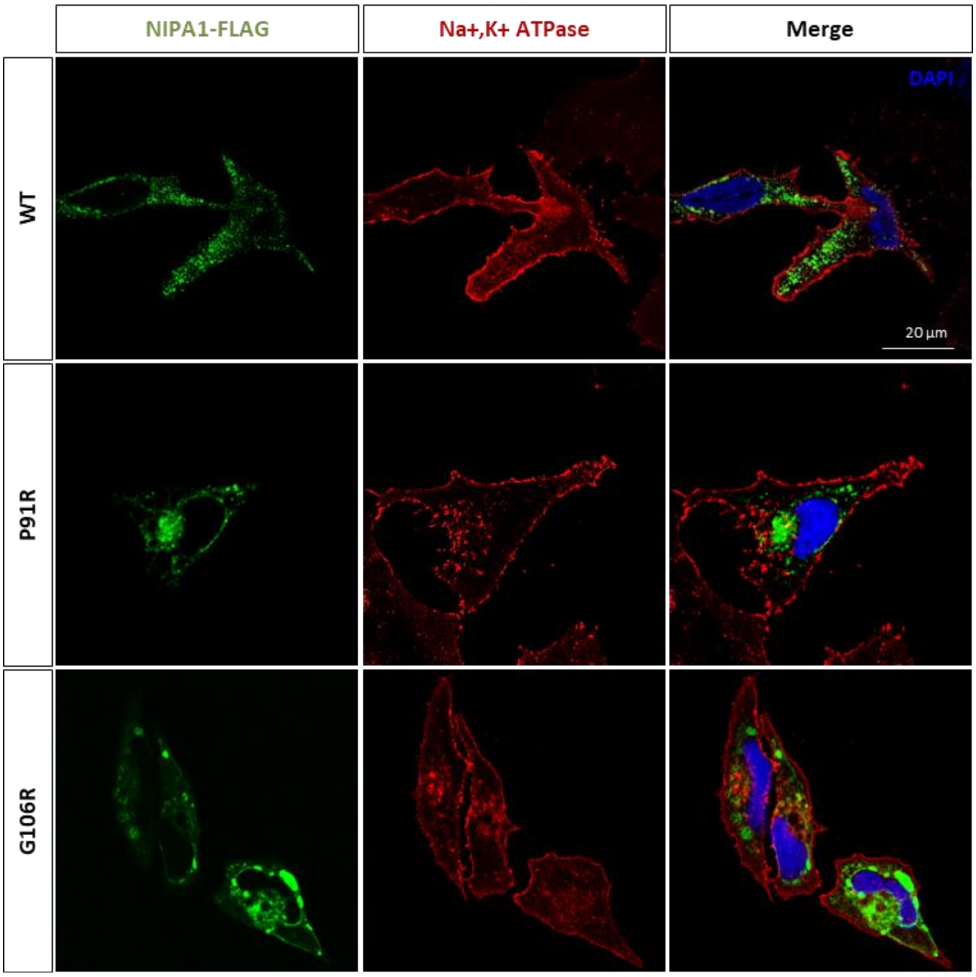

Supplementary Figure S3. Intracellular distribution of transiently expressed wild-type (WT) NIPA1 protein and two NIPA1 mutated forms: p.P91R identified in patient MD-232 and the recurrent clinical mutation p.G106R. HeLa cells were transfected with NIPA1 constructs, tagged with FLAG in C-ter, for 24 hours and subsequently fixed and immunostained using anti-FLAG antibody (green), anti-sodium potassium ATPase (Na^{+},K^{+} ATPase) antibody (red) as a marker of plasma membrane, and DAPI (blue). Representative confocal microscopy images are shown. WT-NIPA1 displays a diffuse cytoplasmatic pattern extended to the cell surface; minimal or no co-localization with Na^{+}, K^{+}ATPase is detected. In contrast, NIPA1-P91R predominantly shows perinuclear distribution and sporadically localizes in larger vesicle-like structures, while NIPA1-G106R is accumulated in large aggregates, as previously described.

Supplementary Table S1. Haplotype constructed for the *PANK2* locus in twelve unrelated carriers of the c.1583C>T (rs137852967) mutation.

Patients ID	Origin	*PANK2* c.1583C>T	rs1078152	*D20S867*	rs6084513	rs137852967	rs6037695	rs7270329	*D20S889*	*D20S116*	rs1628323
			***PANK2* gene**								
MD-002	Spain	HOMOZYGOSIS Roma	A	268	C	**T**	C	G	285	115	T
			A	268	C	**T**	C	G	285	119	T
MD-004			A	268	C	**T**	C	G	285	115	T
			A	268	C	**T**	C	G	285	119	T
MD-007			A	268	C	**T**	C	G	285	115	T
			A	268	C	**T**	C	G	285	119	T
MD-008			A	268	C	**T**	C	G	285	115	T
			A	268	C	**T**	C	G	285	119	T
MD-040			A	268	C	**T**	C	G	285	115	T
			A	268	C	**T**	C	G	285	119	T
MD-101			A	268	C	**T**	C	G	285	115	T
			A	268	C	**T**	C	G	285	119	T
MD-132			A	268	C	**T**	C	G	285	115	T
			A	268	C	**T**	C	G	285	119	T
MD-046	Hungary		C	268	C	**T**	C	G	285	115	T
			C	271	C	**T**	C	G	289	119	T
MD-114	Greece	HETEROZYGOUS Non-Roma	A	268	C	**T**	C	G	285	115	T
			A	270	C	C	C	G	287	109	C
MD-115			A	268	C	**T**	C	G	285	115	T
			A	270	C	C	C	G	287	109	C
MD-112			A	268	C	**T**	C	G	278	103	C
			A	270	C	C	C	G	281	103	C
MD-102	Spain		A	268	A	**T**	T	C	270	109	C
			A	272	A	C	T	C	268	103	C

Supplementary Table S2. Sequencing coverage summary for the 55 samples investigated with the custom gene panel MovDisord.

Patient	Mean Coverage	% Bases							
		>1	>10	>15	>20	>50	>100	>150	>200
**MD-012**	369.93	100	100	100	99.9	99.7	98.7	95.9	90
**MD-018**	424.68	100	100	100	100	99.7	98.6	95.7	89.5
**MD-020**	417.2	100	100	100	100	99.7	98.8	96.5	91.6
**MD-021**	493.46	100	100	100	100	99.8	98.9	97.1	93.4
**MD-023**	368.66	100	100	100	99.9	99.7	98.5	95.2	88.6
**MD-024**	379.66	100	100	100	100	99.6	97.6	93	85.4
**MD-027**	568.79	100	100	100	100	99.8	99.3	98.1	95.9
**MD-037**	353.92	100	100	100	99.9	99.7	98.8	96.7	91.5
**MD-041**	233.64	100	99.9	99.8	99.7	97.2	86	70.7	55.2
**MD-086**	516.01	100	100	100	100	99.8	99	97.2	94.2
**MD-106**	386.23	100	100	100	100	99.7	98	93.9	86.9
**MD-109**	605.06	100	100	100	100	99.8	99.3	98	95.8
**MD-122**	335.85	100	100	100	99.9	99.6	98.3	94	84.9
**MD-126**	397.78	100	100	100	99.9	99.6	97.5	92.8	85.6
**MD-131**	310.47	100	100	100	99.9	99.3	95.8	88.1	76.9
**MD-134**	395.8	100	100	100	99.9	99.7	98.8	96.4	91.3
**MD-137**	390.44	100	100	100	100	99.8	98.5	95.2	88.6
**MD-142**	554.74	100	100	100	100	99.8	99.1	97.6	94.9
**MD-143**	560.46	100	100	100	100	99.8	99.1	97.5	94.6
**MD-148**	400.55	100	100	100	100	99.8	99	96.9	92.2
**MD-153**	376.61	100	100	100	100	99.8	98.7	95.7	89.6
**MD-159**	386.92	100	100	100	100	99.5	97.2	92.1	84.1
**MD-160**	564.34	100	100	100	100	99.8	99.1	97.4	94.5
**MD-161**	527.56	100	100	100	100	99.8	98.8	96.7	93.1
**MD-163**	257.78	100	99.9	99.8	99.7	97.9	88.9	75.5	60.9
**MD-166**	403.67	100	100	100	100	99.8	98.7	96	90.5
**MD-167**	306.19	100	100	100	100	99.6	97.7	91.9	80.8
**MD-168**	393.89	100	100	100	100	99.6	97.4	92.7	85.2
**MD-172**	316.42	100	100	100	100	99.7	98.7	95.2	86.6
**MD-173**	370.92	100	100	100	100	99.7	98.1	93.6	85.2
**MD-174**	233.33	100	99.9	99.9	99.7	97.8	88	72.6	56.5
**MD-176**	356.49	100	100	100	100	99.7	98.6	95.2	88
**MD-179**	388.04	100	100	100	99.9	99.6	97.6	93	85.5
**MD-181**	354.44	100	100	100	100	99.7	98.1	93.6	85.2
**MD-189**	329.95	100	100	100	99.9	99.3	95.7	87.8	77.3
**MD-190**	261.91	100	100	99.9	99.9	99	93.4	81.6	66.8
**MD-193**	359.08	100	100	100	99.9	99.2	95.7	88.5	79.1
**MD-200**	331.37	100	100	100	99.9	99.3	96	88.6	78.3
**MD-206**	259	100	100	99.9	99.8	98.6	91.9	79.9	65.1
**MD-207**	348.49	100	100	100	99.9	99.4	96.6	90.2	81
**MD-208**	354.47	100	100	100	100	99.5	97.1	91.3	82.4
**MD-216**	310.6	100	100	99.9	99.9	98.9	94.1	84.8	73.4
**MD-219**	350.26	100	100	99.9	99.9	99.3	96.2	89.3	80
**MD-228**	355.98	100	100	100	100	99.5	97.1	91.3	82.7
**MD-232**	375.09	100	100	100	100	99.6	97.4	92.6	84.6
**MD-252**	517.62	100	100	100	100	99.7	98.8	96.9	93.8
**MD-255**	592.28	100	100	99.9	99.9	98.9	95.6	91.4	86.7
**MD-270**	557.4	100	100	100	100	99.8	99.3	98	95.7
**MD-273**	559.72	100	100	100	100	99.8	99.2	97.7	95.1
**MD-276**	567.85	100	100	100	100	99.8	99.1	97.7	95
**MD-277**	572.34	100	100	100	100	99.8	99.1	97.5	94.7
**MD-280**	465.06	100	100	100	100	99.8	99.2	97.6	94.4
**MD-281**	509.95	100	100	100	100	99.8	99	97.1	93.7
**MD-429**	391.91	100	100	100	100	99.6	97.6	92.9	85.4
**MD-431**	435.52	100	100	100	100	99.8	98.5	95.4	89.9

Supplementary Table S3. Genes included in the last version of the custom panel MovDisord.

**CLINICAL SIGN (no. genes)**	**517 GENES**
Ataxia (210)	*ABCB7, ACO2, ADCK3, AHI1, ALG6, AMACR, ANO10, APTX, ARL13B, ARSA, ATCAY, ATG5, ATM, ATP1A3, ATP2B3, ATP7A, ATP8A2, ATR, ATXN2, BCKDHA, BCKDHB, BTD, C10ORF2, C5ORF42, CACNA1G, CACNB4, CAMTA1, CASK, CC2D2A, CCDC88C, CEP290, CEP41, CHMP1A, CHP1, CLCN2, CLN5, CLN6, CLP1, COG5, COL18A1, COQ2, COQ4, COQ5, COQ7, COQ9, COX20, CSTB, CTDP1, CTSD, CUL4B, CWF19L1, DAB1, DBT, DCLRE1B, DHTKD1, DKC1, DNAJC19, DNMT1, DYRK1A, EEF2, EIF2B1, EIF2B2, EIF2B3, EIF2B4, EIF2B5, ELOVL4, ELOVL5, EMC1, EP300, EPM2A, ERCC2, ERCC3, ERCC5, ERCC6, ERCC8, EXOSC3, EXOSC8, FAAH2, FAT1, FAT2, FGF14, FLVCR1, FOLR1, FOXC1, GAN, GOSR2, GPR56, GRM1, HEXA, HSD17B4, IFRD1, INPP5E, ITPR1, KCNA1, KCNA2, KCNC3, KCND3, KCNJ10, KCTD7, KDM6A, KIAA0226, KIF26B, KIF7, KMT2D, L2HGDH, LAMA1, LMNB2, MAN2B1, MCEE, MLC1, MMACHC, MMADHC, MORC2, MRE11A, MRPL10, MTFMT, MTTP, NALCN, NHLRC1, NKX2-1, NKX6-2, NOL3, NPC1, OFD1, OPHN1, PARN, PAX6, PC, PCNA, PDHB, PDSS1, PDSS2, PEX10, PEX6, PEX7, PHYH, PIK3R5, PITRM1, PITX2, PLD3, PLP1, PMPCA, PNKP, POLG2, POLR3B, PRICKLE1, PRKCG, PRPS1, PTEN, PUM1, RAD1, RARS2, RIPPLY1, RPGRIP1L, RTEL1, SAMD9L, SCARB2, SCN1A, SCN2A, SCN8A, SCYL1, SEPSECS, SETX, SLC17A5, SLC1A3, SLC25A46, SLC2A1, SLC46A1, SLC52A2, SLC52A3, SLC6A19, SLC9A1, SLC9A6, SNAP25, SNX14, SPTAN1, SPTBN2, SURF1, SYNE1, SYT14, TCTN1, TCTN2, TCTN3, TDP2, TERT, TGFB1, TGM6, THG1L, TINF2, TMEM138, TMEM216, TMEM231, TMEM237, TMEM240, TMEM67, TRAPPC11, TRPC3, TSEN2, TSEN54, TTBK2, TTPA, TTR, UBA5, VHL, VLDLR, VPS53, VRK1, WFS1, XPA, XRCC1*
SPAX (41)	*AARS2, ABHD12, AFG3L2, AIMP1, ARX, CA8, CYP27A1, DARS2, ERLIN1, FAM126A, FXN, GALC, GBA2, GFAP, GJC2, GRID2, KIF1C, L1CAM, LMNB1, LYST, MAG, MARS2, MECP2, MTPAP, OPA1, OPA3, PDYN, PNPLA6, POLR3A, RARS, RPIA, SACS, SIL1, SPG7, STUB1, TPP1, TTC19, VAMP1, VWA3B, WDR73, WWOX*
SPG (69)	*ALDH18A1, ALS2, AMPD2, AP4B1, AP4E1, AP4S1, AP5Z1, ARG1, ARL6IP1, ARSI, ATAD3A, ATL1, B4GALNT1, BICD2, BSCL2, C12ORF65, CAV1, CCT5, CHCHD10, CPT1C, CYP2U1, CYP7B1, DDHD1, DDHD2, DNM2, DSTYK, ENTPD1, EPT1, ERLIN2, FAM134B, FIG4, FLRT1, GAD1, GBE1, GRN, HACE1, HSPD1, IBA57, IFIH1, KIAA0196, KIF1A, KIF5A, KLC2, MARS, NIPA1, NT5C2, PEX16, PGAP1, RAB3GAP2, REEP1, REEP2, RTN2, SARS2, SLC16A2, SPAST, SPG11, SPG20, SPG21, TARDBP, TECPR2, TFG, TRMT5, UBQLN2, USP8, VPS37A, WDR48, ZFR, ZFYVE26, ZFYVE27*
Dystonia (93)	*ACAT1, ANO3, ATP1A2, ATP7B, BCS1L, CACNA1B, CIZ1, COL6A3, COX10, COX15, COX8A, DDC, DLAT, DLD, DNAJC12, EARS2, ECHS1, ETHE1, FARS2, FBXL4, FOXG1, FOXRED1, GCDH, GCH1, GFM1, GFM2, GLB1, GNAL, GNAO1, GTPBP3, HIBCH, HPCA, HPRT1, IARS2, KCTD17, KMT2B, LIAS, LIPT1, LRPPRC, MCOLN1, MICU1, MUT, NARS2, NDUFA1, NDUFA10, NDUFA11, NDUFA12, NDUFA2, NDUFA4, NDUFA9, NDUFAF2, NDUFAF5, NDUFAF6, NDUFS1, NDUFS2, NDUFS3, NDUFS4, NDUFS7, NDUFS8, NDUFV1, NDUFV2, PCCA, PCCB, PDHX, PET100, PNPT1, PRKRA, PTS, QDPR, SCO2, SDHA, SDHAF1, SERAC1, SGCE, SLC19A3, SLC25A19, SLC30A10, SLC39A14, SLC6A3, SPR, SUCLA2, SUCLG1, TACO1, TH, THAP1, TIMM8A, TOR1A, TPK1, TRMU, TSFM, TUBB2B, TUBB4A, UQCRQ*
Choreas (6)	*ADCY5, HTT, PRNP, RNF216, VPS13A, XK*
NBIA (14)	*AP4M1, ATP13A2, C19ORF12, COASY, CP, CRAT, DCAF17, FA2H, FTL, PANK2, PLA2G6, REPS1, SCP2, WDR45*
BGC (5)	*ISG15, PDGFB, PDGFRB, XPR1, SLC20A2*
Parkinson (14)	*CHCHD2, DNAJC13, DNAJC6, FBXO7, GBA, HTRA2, LRRK2, PARK2, PARK7, PINK1, POLG, SNCA, SYNJ1, VPS35*
Others (65)	*ACTB, ADAR, AIFM1, ALDH3A2, AMT, ASPA, ASS1, ATP6AP2, BCAP31, CACNA1A, CAPN1, DCTN1, ETFA, ETFB, ETFDH, FTH1, FUCA1, FUS, GAMT, GATM, GLDC, GLRX5, GTPBP2, HEXB, HTRA1, KIDINS220, KLC4, MECR, MTHFR, NPC2, NUP62, PCSK2, PDE10A, PDE8B, PDHA1, PMM2, PNKD, PPCDC, PPCS, PRRT2, PSEN1, RAB39B, RELN, RNASEH2A, RNASEH2B, RNASEH2C, RNF170, SAMHD1, SCN4A, SLC11A2, SLC25A15, SLC25A42, SLC40A1, SLC6A8, SMPD1, SQSTM1, SUOX, TENM4, TBCD, TBCE, TREX1, TXN2, UCHL1, VCP, WDR81*

SPAX: Spastic Ataxia; SPG: Spastic Paraplegia; NBIA: Neurodegeneration with Brain Iron Accumulation; BGC: Basal Ganglia Calcification
